# Marked Hepatotoxicity Associated With Losartan Treatment: A Case Report

**DOI:** 10.7759/cureus.49846

**Published:** 2023-12-02

**Authors:** Iman Zahedi, Mohamed Sami, Olumide Ijishakin, Henry Onyemarim, Riyotta T Cutliff, Victor C Ezeamii

**Affiliations:** 1 Internal Medicine, Arrowhead Regional Medical Center, San Bernardino, USA; 2 Medicine, American University of Antigua (AUA) College of Medicine, Saint John's, ATG; 3 Surgery, American University of Antigua (AUA) College of Medicine, Saint John's, ATG; 4 Pathology and Laboratory Medicine, Asaba Specialist Hospital, Asaba, NGA; 5 Clinical Sciences, American University of Antigua (AUA) College of Medicine, St. John, ATG; 6 Public Health, Jiann-Ping Hsu College of Public Health, Georgia Southern University, Statesboro, USA

**Keywords:** drug induced hepatotoxicity, angiotensin ii receptor blocker, sub-fulminant hepatitis, hepatotoxicity, losartan

## Abstract

Losartan is a widely prescribed angiotensin II receptor blocker (ARB) used for the management of hypertension and various cardiovascular conditions. While it is generally considered a safe medication, rare cases of hepatotoxicity have been reported in the literature. We present a case of severe hepatic injury and sub-fulminant hepatitis attributed to losartan use in a 54-year-old male patient with underlying hypertension. He presented with a two-week history of abdominal pain, progressive jaundice, dark urine, and vomiting, followed by altered sensorium. His clinical picture, serology, and imaging findings confirmed a severe hepatic injury. After ruling out all possible causes, he was diagnosed with drug-induced hepatotoxicity with losartan treatment. He started improving gradually after losartan discontinuation, N-acetylcysteine administration, and supportive management with close monitoring of liver enzymes. This case report aims to underscore the importance of recognizing losartan as one of the potential causes of hepatotoxicity.

## Introduction

The use of various medications and supplements has surged, contributing to an increased incidence of deranged liver function tests and, in severe cases, frank liver injury. Medications have now emerged as one of the leading causes of acute liver failure in the United States [1]. Although adverse drug reactions associated with medications can affect various organs, these reactions are often reversible and tend to ameliorate upon discontinuation of the offending agent. Losartan, an angiotensin II receptor blocker (ARB), is a drug commonly used in hypertension management and for the prevention of renal damage in patients with diabetes and heart failure [2]. Losartan is generally well-tolerated; however, it can cause a few side effects, including dry cough, weakness, hyperkalemia, anemia, fatigue, or muscle cramps [3]. However, losartan-induced hepatotoxicity, an exceedingly rare adverse effect with high morbidity and mortality, is not widely reported in the literature. Only a few cases have been reported, providing an association between losartan and acute hepatic injury [4]. We report a case of severe hepatic injury attributed to losartan use in a 54-year-old male patient with underlying hypertension.

## Case presentation

A 54-year-old male with a history of hypertension and type 2 diabetes mellitus presented to the emergency department with intermittent severe abdominal pain, yellowish discoloration of the skin, and dark-colored urine for the last 14 days. He also felt dizzy, which prompted a hospital evaluation. Associated symptoms included lethargy, anorexia, intermittent fever, and four episodes of vomiting containing food particles. He was compliant with insulin and was diagnosed with hypertension three months ago, for which he was started on 50mg of losartan daily. He had no history of liver-related diseases or similar diseases. He had no history of smoking, alcohol, or illicit substance abuse.

On initial evaluation, he looked anxious and icteric and was well oriented to time, place, and person. He was afebrile and hemodynamically stable. The abdomen was remarkably tender in the epigastric and right hypochondrium. The rest of the systemic examination was unremarkable. Initial laboratory evaluations revealed remarkably deranged liver function tests (Table 1).

**Table 1:** Results of initial laboratory parameters. ALT: alanine aminotransferase; ALP: alkaline phosphatase; AST: aspartate aminotransferase; INR: international normalized ratio; GGT: gamma-glutamyl transferase

Parameter	Lab values	Reference value
Platelet count	170,000/mm^3^	150,000-350,000
White cell count	9,000/mm^3^	4,000-11,000
ALT	1,641 IU/L	<36
AST	1,425 IU/L	<33
ALP	150 mg/dl	35-95
Total bilirubin	6.1 mg/dl	0.1-1.2
INR	1.7	0.8-1.2
Random glucose level	127 mg/dl	<200
Sodium	136 mEq/l	135-135
Potassium	4.1 mEq/l	3.5-5.5
Calcium	8.9 mg/dl	8.5-10.5
Creatinine	1.1 mg/dl	0.7-1.3
Blood urea nitrogen	22 mg/dl	06-24
Total protein	5.6 g/dl	6.0-8.3
GGT	41 IU/l	<31
Lactate dehydrogenase	323 IU/l	140-280
Prothrombin time	13 seconds	10-13

Due to elevated transaminases, he was immediately commenced on N-acetylcysteine and conservative management with close monitoring of liver enzymes, and losartan was held.

**Figure 1:**
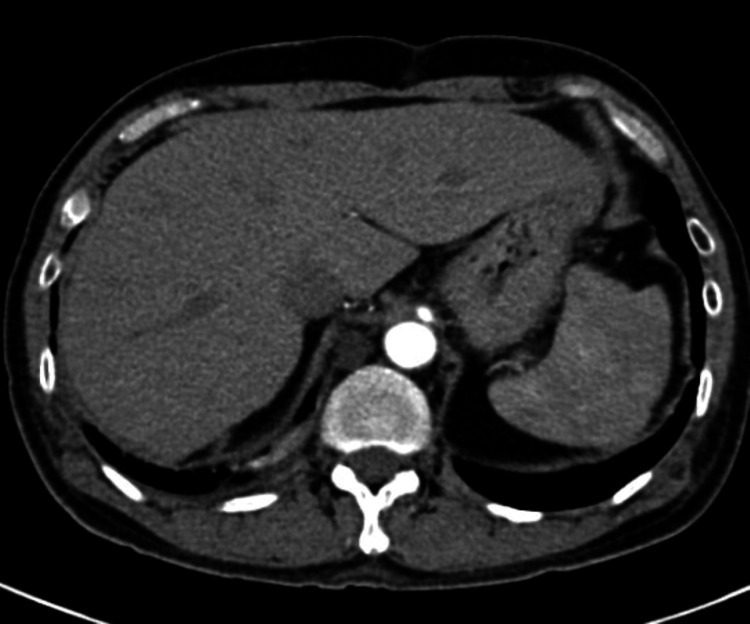
The abdominal CT scan revealed normal liver texture.

He underwent abdominal ultrasonography, which revealed no evidence of biliary obstruction, gallstones, or hepatic steatosis. A CT scan of the abdomen and pelvis revealed no liver abnormalities except for mild steatosis (Figure 1).

Other evaluations included normal pancreatic enzymes and serological tests for viral hepatitis, including hepatitis A, B, and C, which were all negative. Autoimmune liver disease markers, such as antinuclear antibodies (ANA), anti-smooth muscle antibodies (ASMA), and anti-mitochondrial antibodies (AMA), were also within normal limits, ruling out autoimmune hepatitis as a cause. A blood culture was performed to rule out infectious etiology, which was negative. After ruling out all other possible causes, a provisional diagnosis of drug-induced liver injury (DILI) due to losartan was made.

**Figure 2:**
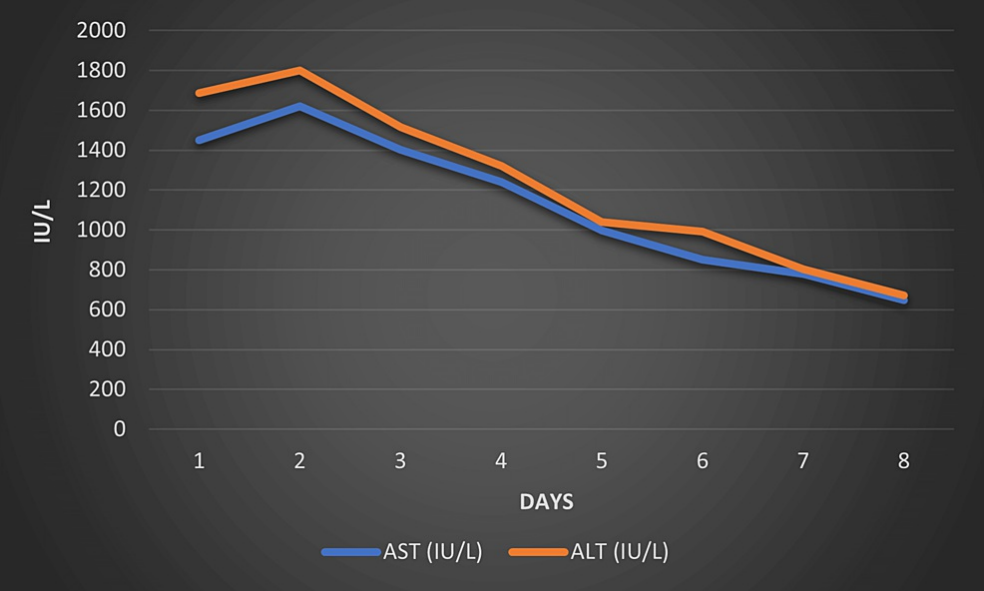
Trend of liver enzymes during the patient's hospital stay AST: aspartate aminotransferase; ALT: alanine aminotransferase

The immediate discontinuation of losartan was recommended, and the patient was managed conservatively with supportive care, including close monitoring of liver function, correction of coagulation abnormalities, and administration of N-acetylcysteine to support hepatic detoxification processes. Over the subsequent weeks, the patient's condition gradually improved. Liver function tests showed a progressive decline in liver enzyme levels, and the jaundice began to recede (Figure 2).

**Figure 3:**
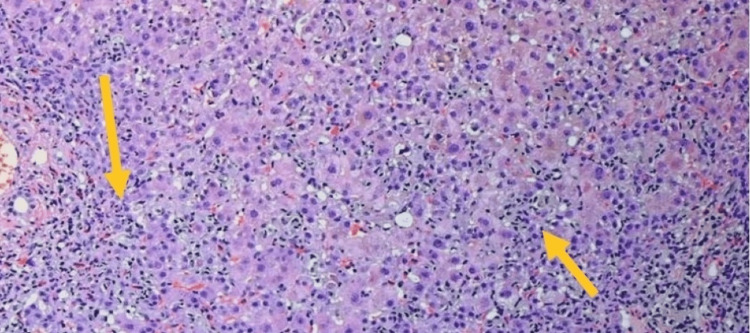
Histopathology of the biopsy demonstrated hepatocellular necrosis with inflammatory infiltrates and minimal cholestasis intrahepatocyte, consistent with drug-induced liver injury.

The patient's clinical symptoms, including fatigue and anorexia, also ameliorated during this period. This favorable response to the withdrawal of losartan and supportive care further strengthened the case for losartan-induced hepatotoxicity. On day seven, a liver biopsy showed marked hepatocellular necrosis with inflammatory infiltrates and moderate or minimal cholestasis intrahepatocyte or intracanalicular, consistent with DILI (Figure 3).

## Discussion

Losartan has been widely recognized for its efficacy in managing hypertension and associated conditions since its approval by the United States Food and Drug Administration (FDA) in 1995 [5]. Despite its extensive use and therapeutic benefits, there have been rare but severe cases of losartan-induced liver injury, which can lead to acute liver failure and necessitate liver transplantation in severe cases [5, 6]. Patients with existing liver conditions should be informed of the risks and advised on the potential need for immediate drug discontinuation and clinical follow-up to prevent severe hepatic damage. The incidence of DILI with losartan as the offending agent is relatively low in the general population (Table 2) [4-9].

**Table 2:** Reported cases of drug-induced liver injury related to losartan. AST: aspartate aminotransferase; ALT: alanine aminotransferase

Author et al.	Age/Sex	Onset of symptoms (days)	AST (IU/L)	ALT (IU/L)	Total bilirubin (mg/dl)	Biopsy	Outcome
Patti et al. [4]	61/F	5	3444	5232	0.7	No	Improved
Al-Halawani et al. [5]	65/F	120	1184	1018	2.7	Yes	Improved
Affes et al. [6]	76/F	30	515	544	5.53	Yes	Died
Diogo et al. [7]	66/M	21	2522	3603	5.28	No	Improved
Andrade et al. [8]	77/M	21	115	410	1.7	No	Improved
Nygaard et al. [9]	55/F	30	635	650	1.3	No	Improved
Nygaard et al. [9]	46/F	90	300	311	NA	No	Improved

The general frequency of liver damage due to losartan is limited, and it has been reported that losartan has been associated with a low rate of serum aminotransferase elevations (<2%) that in controlled trials was no higher than with placebo therapy [10]. However, when it occurs, the damage can manifest in various patterns, such as hepatocellular, cholestatic, or mixed types of liver injury. The diagnosis of DILI is contingent on the examination of liver enzymes, and the pattern of injury can be discerned using the R ratio of serum alanine transaminase (ALT) to serum alkaline phosphatase (ALP) relative to their upper limits of normal [11]. An R ratio of five or more implies hepatocellular injury; two or fewer points toward cholestatic damage; and values between two and five are labeled as mixed [4]. The use of the R ratio is often advised at the initial presentation. A liver biopsy is the gold standard for accurately classifying the type of injury [11]. It is crucial to cease the administration of losartan at the earliest suspicion of DILI, as prompt discontinuation can prevent progression and potentially reverse the liver damage.

The consensus from case studies suggests that losartan-related liver injury is primarily hepatocellular, characterized by a significant rise in transaminases [7]. It is deemed probable when other causes of acute hepatitis are ruled out, and there is a temporal relationship between drug administration and the onset of liver injury. Recovery is possible if losartan is withdrawn promptly. Still, re-exposure can lead to a recurrence of symptoms and liver enzyme elevation, indicating the necessity for patient education to avoid future use of the drug. In some instances, losartan-induced hepatic injury has been irreversible and life-threatening, especially when combined with other medications, such as alendronate, which may potentiate hepatic damage [9].

The pathophysiology of losartan-induced hepatotoxicity remains ambiguous but is presumed to be an idiosyncratic metabolic reaction without typical clinical signs of hypersensitivity [10]. It has been hypothesized that genetic factors affecting the metabolism of ARBs could predispose individuals to hepatotoxicity due to the production of reactive metabolites in the liver. This adverse reaction might also relate to the effects of angiotensin II on the excretion of blood ammonia, particularly in patients with liver cirrhosis and portal hypertension [12,13]. However, age, gender, race, smoking or alcohol, co-administration of other drugs, and host-related and environmental factors may also be involved in drug-related hepatotoxicity [12,14].

Management of losartan-induced liver injury includes holding the offending agent and supportive management, along with close monitoring of the liver function profile [9]. Although losartan-induced liver injury is rare, the published data reveal hepatic injury can occur at any time, from days to months after starting losartan. It is recommended to monitor the dose and concentration of serum losartan levels and liver function enzymes in high-risk patients [11]. Our patient was presented with signs and symptoms of severe hepatic toxicity induced by losartan, and the removal of the offending drug gradually improved his condition.

## Conclusions

Although drug-induced hepatoxicity with losartan has been rarely implicated, it should be considered in patients with unexplained liver injury, and clinicians should remain vigilant, especially during the initial phases of treatment. Prompt recognition, discontinuation of the offending medication, and supportive care are essential for patient management. Our case highlights the critical aspects of losartan-induced hepatic injury, aiming to enhance awareness and improve patient outcomes through careful monitoring and education.
